# Measuring the mutual effects between a CZT detector and MRI for the development of a simultaneous MBI/MRI insert

**DOI:** 10.1186/2197-7364-2-S1-A50

**Published:** 2015-05-18

**Authors:** Ashley Tao, Troy Farncombe, Michael Noseworthy

**Affiliations:** McMaster University, Canada; Hamilton Health Sciences, Canada

While mammography is the gold standard for breast cancer screening, it suffers from poor sensitivity in women with dense breast tissue. Both breast MRI and molecular breast imaging (MBI) have been used as secondary imaging techniques. However, breast MRI suffers from low specificity and low sensitivity in MBI. A CZT based detector system has been developed with the goal of simultaneous MBI/MRI imaging to address the shortcomings of each modality. The performance of each modality needs to be addressed separately and together. The CZT system is comprised of four Redlen CZT modules tiled in a 2x2 array. Each module consists of 256 pixels and feature a builtin on-board ASIC and FPGA. A custom digital readout circuit board was designed to interface the four modules with a microcontroller to a PC. MR images were acquired with a 3T GE Discovery MR750 and Hologic breast coils. A gradient echo imaging sequence was used for all image acquisitions. A tissue mimicking phantom with a plastic grid insert (1 cm spacing) was used to evaluate geometric accuracy with the CZT detectors in the MRI bore. The average distance between the grid markers was 1Å}0.2cm indicating negligible geometric distortion. Field maps were generated with a uniform phantom to quantify the effect on magnetic field homogeneity. Early results indicate a significant distortion (~10ppm) in the magnetic field closest to the coil. Further analysis of the MR images will determine the extent of image quality degradation. A flood map of Tc-99m was acquired to evaluate and implement an energy correction map and a uniformity map. In the absence of a magnetic field, the mean energy resolution at 140keV was 6.3%. After fully characterizing the uniformity, geometric accuracy and sensitivity, the same metrics will be evaluated in the MRI bore.

